# Impact of Therapeutic Interventions on Fear of Progression in Patients with Endometriosis

**DOI:** 10.3390/jcm14103324

**Published:** 2025-05-09

**Authors:** Mircea Adrian Focsa

**Affiliations:** Department of Medical Informatics and Biostatistics, Victor Babes University of Medicine and Pharmacy Timisoara, 2 Eftimie Murgu Sq, 300041 Timișoara, Romania; mfocsa@umft.ro

**Keywords:** endometriosis, fear of progression, anxiety, hormone therapy, surgery

## Abstract

**Background/Objectives**: Endometriosis is a chronic disease associated with pain, infertility, and increased risk of mental disorders (anxiety, depression). One of these manifestations is the fear of progression, recently documented in patients with endometriosis, which can affect their quality of life. Our study aims to evaluate the relationship between the fear of progression and the type of treatment in endometriosis. **Methods**: We conducted a prospective survey of 298 patients with endometriosis, divided into four treatment groups (hormonal therapy without or with surgical indication, surgical intervention, phytotherapy). Fear of progression (FoP) was evaluated through the Fear of Progression Questionnaire—Short Form (FoP-Q-SF). Scores were compared between groups using the Kruskal–Wallis test with Dunn’s post hoc analysis and adjusted for age (ANCOVA). **Results**: FoP was generally high. Significant differences between groups were observed (*p* = 0.021), with the highest FoP-Q-SF scores being in patients undergoing exclusive hormonal treatment, higher than in the surgical groups (*p* < 0.01). Younger age correlated with increased fear (*p* < 0.01). **Conclusions**: Treatment type influences anxiety regarding disease progression. Exclusive hormonal therapy was associated with the greatest fear of progression, while the differences observed between hormonal therapy with surgical indication and primary surgical treatment may partly be due to methodological or informational factors rather than purely clinical differences. A multidisciplinary approach, including psychological support, is essential to alleviate patient fears and improve their quality of life.

## 1. Introduction

Endometriosis is the second most common gynecological condition, affecting about 10% of women of reproductive age [1]. It is characterized by the presence of endometrial-like tissue outside the uterus, triggering chronic inflammation. The main manifestations are chronic pelvic pain and infertility, but the impact of the disease extends beyond the physical domain. Women with endometriosis have an increased risk of mental health disorders—especially anxiety and depression—compared to the general population [2,3]. Chronic pain in endometriosis contributes to dysregulation in neuroendocrine pathways (e.g., involving the hippocampus and amygdala) and is correlated with higher rates of anxiety and depression [4]. These psychological symptoms significantly reduce the patient’s quality of life and can create a vicious cycle in which mental stress exacerbates pain perception and vice versa [5].

One psychological manifestation of chronic illness, noted in endometriosis, is the fear of progression (FoP), which is the persistent fear that the disease will worsen or recur over time. Initially described in cancer patients, fear of progression has recently been documented in women with endometriosis. A 2023 study [6] reported that FoP questionnaire scores are, on average, higher in women with endometriosis than those reported in patients with malignancies (Todd et al., 2023). This specific anxiety about the disease’s course can contribute to insomnia and fatigue and interfere with daily functioning [7]. However, the factors that influence the intensity of the FoP in endometriosis are not fully understood.

We recognize that FoP is closely intertwined with related anxieties such as infertility, repeated surgical interventions, sexual dysfunction, and hysterectomy. Understanding this complexity requires comprehensive patient evaluations addressing all these dimensions.

The relationship between treatment strategy and the patient’s psychological state is a topic of clinical interest. On the one hand, surgery can reduce the physical disease burden by removing lesions, potentially relieving some anxiety related to disease persistence. On the other hand, conservative (hormonal) treatment prolongs disease management without an immediate “end” point, which could leave patients uncertain about the future course of their illness. It is not clear whether certain therapeutic approaches can diminish or, conversely, amplify the fear of progression. Patient age or previous experiences may also modulate how individuals perceive the risk of disease worsening.

This study aimed to evaluate the impact of different therapeutic interventions on the level of fear of progression in endometriosis. We compared the degree of concern about disease evolution (as measured by the Fop-Q-SF score) among patients in four distinct treatment groups, defined by their management strategy (hormonal, surgical, or combined).

## 2. Materials and Methods

### 2.1. Study Design and Participants

This prospective observational study was conducted at Endoinstitut–Regina Maria Hospital, Timisoara, from 1 December 2023, to 30 March 2024. We enrolled adult women (≥18 years) with a confirmed diagnosis of endometriosis who provided informed consent. Endometriosis was diagnosed either laparoscopically or via imaging. In patients who had not undergone surgery, diagnosis was established via characteristic findings on transvaginal ultrasound (e.g., ovarian endometriomas, deep infiltrating nodules), or on pelvic MRI when ultrasound results were inconclusive or suggested extensive disease. For those who underwent operative treatment, laparoscopic visualization and histopathology confirmed the diagnosis. A total of 298 patients meeting these criteria were included. Based on their treatment plan at inclusion, they were grouped into four cohorts, as follows:

- Group 1—Hormonal Therapy Only: Patients receiving exclusive hormonal therapy for endometriosis, with no current surgical intervention planned. Group 1 represents those managed conservatively without an imminent need for surgery.
- Group 2—Hormonal Therapy + Planned Surgery: Patients on hormonal therapy who also had a definite indication for surgery and were awaiting a surgical procedure. In this cohort, hormonal treatment was a provisional or bridging therapy to control symptoms until surgery could be performed. Planned surgeries included laparoscopic excision or ablation of endometriosis lesions, for example, cystectomy for ovarian endometriomas, resection of deep infiltrating endometriotic nodules, and adhesiolysis. Patients receiving medical therapy before surgery may reflect a clinical strategy or logistical considerations, such as surgical waiting lists. This potential heterogeneity should be considered when interpreting results.
- Group 3—Primary Surgical Management: Patients managed with surgical intervention for endometriosis without concurrent hormone therapy. This group included individuals who proceeded directly to surgery as a first-line treatment due to either the severity of the disease or contraindications/intolerance to hormonal therapy. It also included a few patients who had initially tried hormones but discontinued them (due to side effects or personal choice) and opted for surgery.
- Group 4—Phytotherapy/Alternative Therapy: Patients who refused hormonal therapy and instead pursued alternative treatments for endometriosis, with no surgical treatment received to date. These patients declined standard hormonal management due to personal preference or contraindications and were managing their symptoms with approaches such as phytotherapy (herbal and nutritional treatments) and lifestyle modifications.

### 2.2. Procedures and Instruments

At the time of inclusion, all participants completed a form that included demographic data (age, history of the disease) and the Fear of Progression Questionnaire—Short Form (FoP-Q-SF) [8]. This validated tool for chronic diseases includes 12 items on concerns about the course of the disease, each rated on a 5-point Likert scale (1—“never”; 5—“very often”). A total score (sum or average) is calculated, with higher values indicating a more pronounced fear of disease progression. We also recorded responses to each item of the FoP-Q-SF questionnaire to identify the most intense specific areas of concern (e.g., fear of pain, concern about effects on family, body image, etc.).

### 2.3. Data Analysis

Data collected and curated in Microsoft Excel (Office 2021) was further analyzed using the open-source JASP (v. 0.19.1.0) application. A descriptive analysis of patients’ baseline characteristics and FoP-Q-SF scores was performed in each group (means, standard deviation, item medians). Group comparability was assessed by appropriate tests (ANOVA or nonparametric equivalent) for key demographic variables. As Levene’s test showed an uneven variance of FoP-Q-SF scores between groups (*p* = 0.01), a non-parametric Kruskal–Wallis test was chosen to compare the distributions of total FoP-Q-SF scores between the four groups. In case of significant overall difference, Dunn’s post hoc test for pair comparisons was applied, with adjustment of the significance level via Bonferroni correction. To assess the potential influence of age on the observed differences, a covariance analysis (ANCOVA) was used, with age as the covariate, with the FoP-Q-SF score as the dependent variable, and the treatment group as an independent factor. The threshold of statistical significance was set at α = 0.05.

## 3. Results

### 3.1. Participant Characteristics

**Figure 1:**
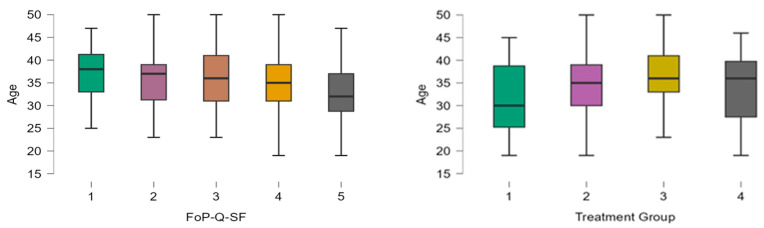
The age distribution of patients across Fear of Progression Questionnaire—Short Form (FoP-Q-SF) scores and treatment groups.

A total of 298 patients were included, with 34 in Group 1 (Hormone only), 128 in Group 2 (Hormone + Surgery), 122 in Group 3 (Surgery), and 14 in Group 4 (Phytotherapy). The mean age of the entire cohort was 34.9 ± 6.4 years, and ages differed slightly by group; Group 1 patients were younger on average (mean ~32 years), while Group 3 patients were older (mean ~36 years). Thus, patients with surgical indications and no hormonal treatment tend to be older on average compared to those with hormonal treatment and no surgical indication, who are the youngest. The analysis of patient age distribution across different FoP-Q-SF scores revealed an inverse relationship between age and fear of progression, with younger patients consistently reporting higher FoP scores (Figure 1). Specifically, patients exhibiting the highest level of fear (FoP score = 5) had a mean age of approximately 32.6 years, while those reporting the lowest fear levels (FoP score = 1) had a mean age of 37.5 years. This finding emphasizes younger age as a significant factor associated with increased anxiety regarding disease progression.

The difference between the lowest and highest average scores between treatment groups was relatively small, with all groups being above the level considered problematic in the literature (score ≥ 34 out of 60, equivalent to 2.8 out of 5) [9].

The distribution of patients among treatment groups is not uniform; most belong to Group 2 (128, 42.9 %) and Group 3 (122, 40.9%).

**Figure 2:**
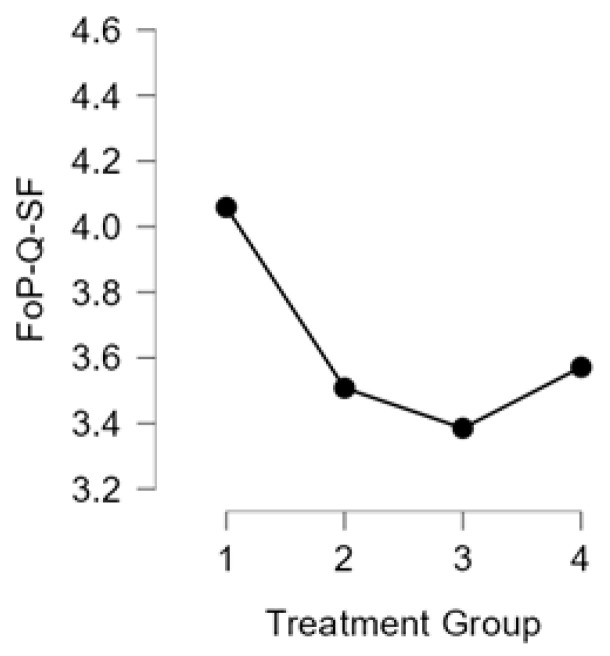
The distribution of FoP-Q-SF scores across treatment groups.

Regarding FoP-Q-SF scores, the level of fear of progression was high overall, with a median across the entire study population of 4 (on a scale of 1–5), corresponding to a moderate–high level of concern. Comparing between groups, Group 1 (Hormone only) recorded the highest mean FoP-Q-SF SCORE (4.06), followed by Group 4 (3.57) and Group 2 (3.51). Group 3 had the lowest average level of fear (3.38) (Figure 2).

Analysis of individual items in the questionnaire revealed that the most intense concerns among patients were fear of medical procedures (e.g., anxiety before an intervention or check-up) and the impact of the disease on body image (concerns about physical changes caused by the disease or treatment). Both had a median response of 4 (“often”) out of 5, indicating a high level of concern. In contrast, concern that the disease could be passed on to children (heredity concern) was the lowest, with a median of 2 (“rare”). Other aspects, such as fear of future chronic pain or decreased performance at work, had intermediate levels of concern, with medians between 3 and 4.

### 3.2. Comparison of Scores Between Groups

The Kruskal–Wallis test applied to the FoP-Q-SF total scores indicated statistically significant differences between the four treatment groups (χ^2^ = 9.7, *p* = 0.021). This result suggests that at least one group differs from the others in terms of the level of fear of progression. The post hoc analysis (Dunn’s with Bonferroni correction) showed that Group 1 (hormonal, non-surgical) has a significantly higher FoP-Q-SF score than Group 3 (surgical, non-hormonal), with a median difference of ~0.7 points on a scale of 1–5 (*p* = 0.002). Group 1 also has significantly higher scores than Group 2 (hormonal + surgical, with a median difference of ~0.4 (*p* = 0.014)). No other comparison between pairs of groups reached the threshold of statistical significance (i.e., the differences between Group 2 vs. Group 3, 1 vs. 4, 2 vs. 4, and 3 vs. 4 were not significant).

These results indicate that patients under hormone treatment alone, without planned surgery, have higher levels of fear of progression than patients who are being considered or have undergone surgery. The most pronounced difference was between the hormone-only treatment group and the surgical approach group without hormone therapy, where the fear level was significantly lower.

### 3.3. Adjusted Analysis (ANCOVA)

Given that the groups showed age differences, we also performed an ANCOVA analysis to adjust the FoP-Q-SF scores according to age. Including age as a control variable, the differences between treatment groups remained significant. In the ANCOVA model, belonging to Group 1 (hormone treatment only) was associated with a significantly higher FoP-Q-SF score compared to Group 2 (coefficient of −0.48, *p* = 0.028) and Group 3 (coefficient of −0.54, *p* = 0.014). This confirms that Group 2 and Group 3 have significantly lower scores than Group 1, even after age adjustment. Age itself had a significant negative association with the FoP-Q-SF score (coefficient −0.03 per year, *p* = 0.004), indicating that, regardless of the treatment group, younger patients tend to have a higher fear of progression than older ones.

In summary, the quantitative results emphasize two main aspects: (1) the type of treatment followed in endometriosis is associated with differences in the level of fear of progression, with hormone-only therapy being linked to more significant anxiety about the evolution of the disease; (2) the age of the patients is inversely proportional to the intensity of this fear, with the younger patients being the more vulnerable group from this point of view.

## 4. Discussion

The present study highlights how specific clinical management factors can influence the psychological state of patients with endometriosis. We found that patients undergoing hormone therapy alone (Group 1) exhibited higher levels of fear of progression compared to patients who had surgical treatment (Group 2 and Group 3). In contrast, patients in the surgical group (Group 3), who had operative intervention without ongoing medical therapy, had significantly lower fear of progression scores. Patients receiving combined therapy or awaiting surgery (Group 2) and those pursuing alternative therapies (Group 4) had intermediate FoP levels. These results suggest that the absence of an immediate surgical “solution” may be associated with heightened anxiety about the future of the disease, whereas having a definitive plan for surgery (or having had surgery) can provide some psychological relief by reducing uncertainty.

The fear of progression observed in patients with endometriosis may be influenced not only by disease pathology but also by the quality and consistency of information provided by healthcare professionals. Variability in patient counseling, clarity of communication, and perceptions of medical competence might significantly shape patient fears, highlighting the necessity for standardized, clear, and empathetic patient education.

In endometriosis management, surgery (typically laparoscopic excision of lesions) is often perceived by patients as a crucial step towards symptom relief or even a form of “temporary cure”. Our finding that Group 3 (surgery) patients had much lower FoP levels than those in Group 1 supports the idea of a beneficial psychological effect of surgical intervention or the prospect thereof. Patients who know their disease is being addressed directly, by removing lesions, might feel less worried that the condition will silently worsen, thereby reducing FoP. By contrast, patients in Group 1, who rely solely on medical therapy, may harbor concerns that while on hormones, the disease is merely suppressed, not eliminated; thus, the risk of progression persists. This lingering uncertainty can fuel anxiety. In our study, even Group 2 patients (on hormones but with a planned surgery) tended to have lower FoP than those on hormones without a plan for surgery, underscoring the reassurance conferred by a concrete surgical plan. It should be noted that differences or the lack thereof observed between Group 2 (hormonal therapy with planned surgery) and Group 3 (primary surgical treatment) might be at least partially attributable to methodological artefacts, including differences in patient counselling, information received, and the timing of intervention.

Aside from treatment modality, age emerged as an important factor associated with FoP. Younger patients had a more pronounced fear of progression than older patients. This observation is in line with findings in oncology, where younger cancer patients often report greater fear of recurrence/progression than older patients [10]. In the context of endometriosis, younger women—often in the early reproductive stage and frequently nulliparous—may be particularly worried about how the disease could impact their fertility and life plans (career, family) in the long run. These concerns can manifest as elevated FoP. Conversely, as patients become older, they may develop better coping mechanisms or reach life milestones that reduce uncertainty (for example, completing their families), which can diminish disease-related fears. Also, older patients might simply have had more time to come to terms with chronic illness, leading to lower anxiety about its progression.

Our results confirm that fear of progression is a significant issue for many endometriosis patients, and they align with the emerging literature on this topic. For example, Todd et al. (2023) [6] reported high FoP scores in women with endometriosis, even higher than those observed in cancer patients. Similarly, a recent study by Pickup et al. (2024) [7] found that fear of progression in endometriosis contributes to fatigue and sleep disturbances, especially when paired with pain and depression. These findings across studies indicate that FoP is not just an incidental psychological symptom; it can amplify the overall burden of the disease by exacerbating insomnia, fatigue, and perhaps pain perception.

From a clinical standpoint, our findings suggest that identifying subgroups of patients at higher risk of elevated FoP—in our case, patients managed exclusively with medical therapy and younger patients—is important for providing targeted support. Awareness of a patient’s fear of progression can prompt healthcare providers to take proactive steps. First, effective communication is crucial. For patients on medical treatment without immediate surgery, a clear explanation of the treatment plan and prognosis might alleviate uncertainty. Explaining why a conservative approach is being used, what the backup options are (including surgery if needed in the future), and acknowledging the patient’s fears can help reduce feelings of helplessness. We have observed that when physicians explicitly discuss the rationale and contingencies of management plans, patients often feel more secure and less anxious. In contrast, poor communication or not addressing the patient’s concerns may leave them feeling “in limbo”, which can intensify FoP [11]. Secondly, integrating psychological support into the care of endometriosis patients could be beneficial, particularly for those with high FoP. Interventions such as psychological counseling or cognitive–behavioral therapy (CBT) can help patients develop coping strategies to manage anxiety about the future. Mind–body techniques like mindfulness meditation, yoga, and relaxation exercises have shown promise in reducing stress and improving pain outcomes in endometriosis [12]. While more research is needed to confirm their efficacy, involving mental health professionals as part of a multidisciplinary endometriosis team may help address the fear of progression directly. For instance, a psychologist or counselor can work with patients to challenge catastrophic thoughts about disease progression and to maintain a hopeful outlook alongside realistic monitoring of symptoms.

It is noteworthy that patients in Group 4 (phytotherapy) did not report significantly different FoP levels compared to the other groups, despite not using conventional treatments. Their FoP-Q-SF scores were moderate (and quite variable between individuals). This could indicate that there are individual personality or belief factors at play that modulate fear independently of treatment type. For example, some patients in Group 4 might have refused hormones due to fear of side effects or a strong preference for “natural” therapies, but this does not necessarily mean they are more fearful about the disease progression itself; in fact, such patients might be very proactive and feel empowered by taking alternative measures, thereby keeping their FoP in check. Others in Group 4 might actually have high FoP but simultaneously high skepticism toward conventional medicine, leading them to seek control via alternative means. Without additional qualitative data on why each Group 4 patient chose phytotherapy, these interpretations remain speculative. What this group does illustrate is the heterogeneity of patient coping styles: fear of progression can be high or low regardless of treatment path, possibly influenced by personal attitudes, understanding of the disease, and trust in treatments.

Limitations: The main limitation of this study is its cross-sectional design, which does not allow us to establish causation between treatment type and FoP. We cannot exclude the possibility of selection bias; for instance, it could be that patients with higher anxiety about their disease gravitated toward (or were steered toward) a particular treatment modality. We attempted to mitigate this by noting that treatment decisions were primarily driven by clinical indications. Another limitation is that we did not systematically assess certain clinical factors that might influence FoP, such as the severity of chronic pain, the precise surgical stage of endometriosis, or the presence of other mental health comorbidities (like pre-existing generalized anxiety disorder). We recognize that disease “stage” could affect a patient’s outlook (for example, patients knowing they have an advanced disease might feel more anxious). Additionally, the relationship between pelvic pain and endometriosis is complex, as pelvic pain in patients diagnosed with endometriosis may not always be directly attributable to the disease itself. This underscores the need for precise diagnostic criteria and thorough clinical assessments in future research.

We did not follow patients over time in this study. It remains unknown as to how a patient’s fear of progression might evolve after treatment; for example, does FoP decrease after a successful surgery, or does it potentially increase if symptoms recur or if a patient stays long-term on medical therapy? Our findings provide a snapshot, but prospective longitudinal studies are needed to observe changes in FoP with time and treatment. Such studies could also clarify whether intervening on FoP (through counseling or otherwise) leads to better patient outcomes.

## 5. Conclusions

This study highlights that fear of progression is common among patients with endometriosis and varies depending on the therapeutic approach. Those treated with hormone therapy alone have the highest levels of fear about the evolution of the disease, while patients who undergo surgery (or are considering an intervention) tend to be less anxious from this perspective. Young patients are also more susceptible to progression anxiety compared to older patients.

These findings underscore the need for an integrated approach to endometriosis management that addresses both the physical and emotional components. In addition to treating injuries and relieving somatic symptoms, it is necessary to support patients through counselling and interventions aimed at reducing anxiety and increasing the feeling of control. Multidisciplinary care—involving gynecologists, surgeons, psychologists, and pain medicine specialists—is ideal for breaking the vicious circle between pain and mental suffering and improving quality of life. By recognizing and addressing the fear of progression, we can provide truly patient-centred care aligned with the principles of personalized and humanistic medicine in endometriosis.

## Data Availability

The authors will make the raw data supporting this article’s conclusions available upon request.
